# Supplementary material for: Evaluating the Turkish validity and reliability of the Brief Illness Perception Questionnaire in periodontal diseases
Source: PeerJ. 2023 Sep 11;11:e16065. doi: 10.7717/peerj.16065 (PMC10501379; doi:10.7717/peerj.16065)
Supplement: Supplemental Information 3 [file peerj-11-16065-s003.docx]

| **Kısa Hastalık Algısı Ölçeği**  Aşağıdaki sorular için görüşünüzü en iyi yansıtan rakamı yuvarlak içerisine alınız.  1. Hastalığınız hayatınızı ne kadar etkiliyor?  0 1 2 3 4 5 6 7 8 9 10  Hiç Hayatımı  etkilemiyor şiddetli şekilde  etkiliyor  2. Sizce hastalığınız ne kadar devam edecek?  0 1 2 3 4 5 6 7 8 9 10  Çok kısa Sonsuza dek  süre  3. Hastalığınız üzerinde ne kadar kontrole sahip olduğunuzu hissediyorsunuz?  0 1 2 3 4 5 6 7 8 9 10  Kesinlikle Aşırı  kontrolsüzüm derecede  kontrollüyüm  4. Tedavinizin hastalığınıza ne kadar yardımcı olabileceğini düşünüyorsunuz?  0 1 2 3 4 5 6 7 8 9 10  Hiçbir Son derece  şekilde yardımcı yardımcı  olmayacağını düşünüyorum olabileceğini düşünüyorum  5. Hastalığınızın belirtilerini ne kadar hissediyorsunuz?  0 1 2 3 4 5 6 7 8 9 10  Hiçbir Birçok  belirti şiddetli  hissetmiyorum belirti hissediyorum  6. Hastalığınız hakkında ne kadar endişelisiniz?  0 1 2 3 4 5 6 7 8 9 10  Hiç Son derece  endişeli değilim endişeliyim  7. Hastalığınızı ne kadar iyi anlayabildiğinizi düşünüyorsunuz?  0 1 2 3 4 5 6 7 8 9 10  Hiç Çok iyi  anlamıyorum anlıyorum  8. Hastalığınız sizi duygusal olarak ne kadar etkiliyor? (Örneğin, sizi kızdırıyor mu, korkutuyor mu, üzüyor mu ya da depresyona mı sokuyor?)  0 1 2 3 4 5 6 7 8 9 10  Duygusal olarak Duygusal olarak  hiç etkilemedi çok etkiledi    9. Lütfen hastalığınıza neden olduğuna inandığınız en önemli üç faktörü sıralayınız.  Benim için en önemli sebepler: |
| --- |
