# Supplementary material for: Evaluating the Turkish validity and reliability of the Brief Illness Perception Questionnaire in periodontal diseases
Source: PeerJ. 2023 Sep 11;11:e16065. doi: 10.7717/peerj.16065 (PMC10501379; doi:10.7717/peerj.16065)
Supplement: Supplemental Information 4 [file peerj-11-16065-s004.pdf]

Hastane Anksiyete ve Depresyon Ölçeği (HAD)

(Hospital Anxiety and Depression Scale (HADS))

Hastanın Adı Soyadı: ______________________________________ Tarih: ___/___/______

Her maddeyi okuyun ve son birkaç gününüzü göz önünde bulundurarak nasıl hissettiğinizi en iyi ifade eden yanıtın yanındaki kutuyu işaretleyin. Yanıtınız için çok düşünmeyin, aklınıza ilk gelen yanıt en doğrusu olacaktır.

1. Kendimi gergin "patlayacak gibi" hissediyorum.

- ❑$_3$ Çoğu zaman
- ❑$_1$ Zaman zaman, bazen
- ❑$_2$ Birçok zaman
- ❑$_0$ Hiçbir zaman

2. Eskiden zevk aldığım şeylerden hala zevk alıyorum.

- ❑$_0$ Aynı eskisi kadar
- ❑$_2$ Yalnızca biraz eskisi kadar
- ❑$_1$ Pek eskisi kadar değil
- ❑$_3$ Hiçbir zaman

3. Sanki kötü bir şey olacakmış gibi bir korkuya kapılıyorum.

- ❑$_3$ Kesinlikle öyle ve oldukça da şiddetli
- ❑$_2$ Evet, ama çok da şiddetli değil
- ❑$_1$ Biraz, ama beni pek endişelendirmiyor
- ❑$_0$ Hayır, hiç de öyle değil

4. Gülebiliyorum ve olayların komik tarafını görebiliyorum.

- ❑$_0$ Her zaman olduğu kadar
- ❑$_2$ Kesinlikle o kadar değil
- ❑$_1$ Şimdi pek o kadar değil
- ❑$_3$ Artık hiç değil

5. Aklımdan endişe verici düşünceler geçiyor.

- ❑$_3$ Çoğu zaman
- ❑$_1$ Zaman zaman, çok sık değil
- ❑$_2$ Birçok zaman
- ❑$_0$ Yalnızca bazen

6. Kendimi neşeli hissediyorum.

- ❑$_3$ Hiçbir zaman
- ❑$_1$ Bazen
- ❑$_2$ Sık değil
- ❑$_0$ Çoğu zaman

7. Rahat rahat oturabiliyorum ve kendimi rahat hissediyorum.

- ❑$_0$ Kesinlikle
- ❑$_2$ Sık değil
- ❑$_1$ Genellikle
- ❑$_3$ Hiçbir zaman

8. Kendimi sanki durgunlaşmış gibi hissediyorum.

- ❑$_3$ Hemen hemen her zaman
- ❑$_1$ Bazen
- ❑$_2$ Çok sık
- ❑$_0$ Hiçbir zaman

Acta psychiatr. scand. 1983:67:361-370 A. S. Zigmond and R. P. Snaith

9. Sanki içim pır pır ediyormuş gibi bir tedirginliğe kapılıyorum.

- ❑$_0$ Hiçbir zaman
- ❑$_2$ Oldukça sık
- ❑$_1$ Bazen
- ❑$_3$ Çok sık

10. Dış görünüşüme ilgimi kaybettim.

- ❑$_3$ Kesinlikle
- ❑$_2$ Gerektiği kadar özen göstermiyorum
- ❑$_1$ Pek o kadar özen göstermeyebilirim
- ❑$_0$ Her zamanki kadar özen gösteriyorum

11. Kendimi sanki hep bir şey yapmak zorundaymışım gibi huzursuz hissediyorum.

- ❑$_3$ Gerçekten de çok fazla
- ❑$_1$ Çok fazla değil
- ❑$_2$ Oldukça fazla
- ❑$_0$ Hiç değil

12. Olacakları zevkle bekliyorum.

- ❑$_0$ Her zaman olduğu kadar
- ❑$_1$ Her zamankinden biraz daha az
- ❑$_2$ Her zamankinden kesinlikle daha az
- ❑$_3$ Hemen hemen hiç

13. Aniden panik duygusuna kapılıyorum.

- ❑$_3$ Gerçekten de çok sık
- ❑$_1$ Çok sık değil
- ❑$_2$ Oldukça sık
- ❑$_0$ Hiçbir zaman

14. İyi bir kitap, televizyon ya da radyo programından zevk alabiliyorum.

- ❑$_0$ Sıklıkla
- ❑$_2$ Pek sık değil
- ❑$_1$ Bazen
- ❑$_3$ Çok seyrek

Mavi renkli kutu içinde şıkları olan sorular anksiyete, turuncu renkli altı çizgili şıkları olan sorular depresyon skorlarını verir.

0-7 puan: normal ||| 8-10puan: sınırda ||| 11ve üstü anormal

Toplam Puan: Depresyon _________ Anksiyete ________

ftronline
www.ftronline.com

Tasarım ve düzenleme: Dr. Ender Salbaş 2018
